# Anchor Side Chains of Short Peptide Fragments Trigger Ligand-Exchange of Class II MHC Molecules

**DOI:** 10.1371/journal.pone.0001814

**Published:** 2008-03-19

**Authors:** Shashank Gupta, Sabine Höpner, Bernd Rupp, Sebastian Günther, Katharina Dickhaut, Noopur Agarwal, M. Cristina Cardoso, Ronald Kühne, Karl-Heinz Wiesmüller, Günther Jung, Kirsten Falk, Olaf Rötzschke

**Affiliations:** 1 Max-Delbrück-Center for Molecular Medicine (MDC), Berlin, Germany; 2 Leibniz-Institute for Molecular Pharmacology (FMP), Berlin, Germany; 3 Charite Berlin, Berlin, Germany; 4 University of Tübingen, Tübingen, Germany; Federal University of São Paulo, Brazil

## Abstract

Class II MHC molecules display peptides on the cell surface for the surveillance by CD4+ T cells. To ensure that these ligands accurately reflect the content of the intracellular MHC loading compartment, a complex processing pathway has evolved that delivers only stable peptide/MHC complexes to the surface. As additional safeguard, MHC molecules quickly acquire a ‘non-receptive’ state once they have lost their ligand. Here we show now that amino acid side chains of short peptides can bypass these safety mechanisms by triggering the reversible ligand-exchange. The catalytic activity of dipeptides such as Tyr-Arg was stereo-specific and could be enhanced by modifications addressing the conserved H-bond network near the P1 pocket of the MHC molecule. It affected both antigen-loading and ligand-release and strictly correlated with reported anchor preferences of P1, the specific target site for the catalytic side chain of the dipeptide. The effect was evident also in CD4+ T cell assays, where the allele-selective influence of the dipeptides translated into increased sensitivities of the antigen-specific immune response. Molecular dynamic calculations support the hypothesis that occupation of P1 prevents the ‘closure’ of the empty peptide binding site into the non-receptive state. During antigen-processing and -presentation P1 may therefore function as important “sensor” for peptide-load. While it regulates maturation and trafficking of the complex, on the cell surface, short protein fragments present in blood or lymph could utilize this mechanism to alter the ligand composition on antigen presenting cells in a catalytic way.

## Introduction

The endosomal route is considered to be the default pathway for the loading of class II MHC molecules. Here the MHC molecule encounters internalized proteins serving as antigen source inside the cell in an acidic lysosomal-like compartment (MIIC) [1], [2]. However, experiments with fixed cells or MHC expressing cells lacking key components of the processing pathway indicate that MHC loading can take place also directly on the cell surface. This applies not only for optimally sized peptides but also for larger polypeptide chains or even full-length proteins [3]–[5]. In particular immature dendritic cells (DC) could utilize this pathway. These DC contain a large fraction of ‘empty’ class II MHC molecules on the cell surface, which may allow the direct capturing of antigens from the extracellular space [6], [7].

While for these cells cell-surface processing seems to represent a major antigen-loading pathway, on other cells it could cause irregular immune responses. Namely on mature DC or activated B cells the ligands presented on the cell surface should accurately reflect the peptide composition inside the MIIC compartment. Presumably as safeguard, a mechanism has evolved that prevents the ‘accidental’ loading of class II MHC molecules. MHC molecules, once they have lost their ligand, rapidly convert into a stable inactive state that is ‘non-receptive’ for free peptides [8], [9]. Neither the receptive nor the non-receptive conformation has been structurally defined yet, so that they are characterized solely by their kinetic parameters.

While in principle the conversion between the two forms is reversible, the equilibrium is largely shifted towards the non-receptive conformation. As a consequence, cell surface loading is usually very inefficient, which hinders the induction of productive immune responses during peptide vaccinations. In the endosomal processing pathway MHC-loading is facilitated by HLA-DM, a chaperone stabilizing the ‘peptide receptive’ state [10], [11]. In previous studies we have shown that surprisingly also small organic compounds can exhibit this effect [12], [13]. Similarly to HLA-DM, these ‘MHC-loading enhancers’ (MLE) stabilize a peptide receptive state resulting in accelerated antigen-loading and ligand exchange. Here we demonstrate that not only simple organic chemicals but also amino acid side chains can mediate this effect. In experiments with short dipeptides we could demonstrate that they can provoke both ligand exchange and peptide loading when targeted to the conserved P1 anchor pocket of the class II MHC molecule. The presence of ‘peptide-MLE’ during T cell assays therefore significantly improved the cell surface loading of antigen presenting cells (APC) with peptide antigens, which directly translated into an increased sensitivity of antigen-specific T cell responses.

## Results

**Figure 1:**
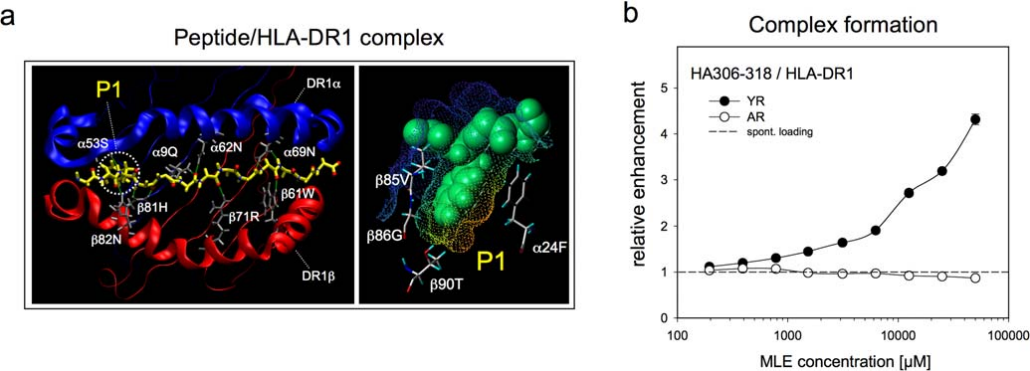
Amino acid side chains of short peptide fragments can catalyze the formation of MHC/ligand complexes. a) Location of P1 and the H-bond network in class II MHC molecules. Left panel: top view on the peptide binding site of the class II MHC molecule HLA-DR1. Only the α_1_- (blue ribbon) and the β_1_-domain of the MHC molecule (red ribbon) are depicted; position of P1 is indicated. The backbone of the peptide ligand HA306-318 and the anchor side chain filling the P1 pocket are shown in yellow; MHC residues forming H-bonds with the backbone are labelled in grey. Right panel: side view of a P1 pocket loaded with the tyrosine anchor side chain. Surface of the pocket is indicated in yellow; amino acid residues forming this pocket are indicated; the peptide is shown in spacefill mode (green). Images are based on the crystal structure of HA306-318/HLA-DR1 (PDB: 1DLH) [38]. b) The catalytic impact of dipeptide side chains on the formation of antigen-complexes. The influence of short peptides on the complex formation-rate between HA306-318 and soluble HLA-DR1 was determined. The loading reaction was carried out in the presence of titrated amounts of the dipeptides Tyr-Arg (YR, filled circle) or Ala-Arg (AR, open circle) or in the absence of these dipeptides (dashed line). Complex formation was determined by ELISA and is expressed as relative enhancement in reference to the spontaneous complex formation in the absence of any catalyst. The amount of catalytic peptide fragments (MLE concentration) is indicated on the x-axis.

Studies with synthetic organic MHC ligand-exchange catalysts pointed already to the P1-pocket as specific target site for ‘MHC-loading enhancer’ (MLE) [12]. The pocket is located in the binding groove close to the N-terminal side of the peptide ligand. In the ligand-complex the pocket accommodates the side chain of a key anchor residue of the peptide (Fig. 1A). It was therefore assumed that the MLE-effect may also be achieved with small natural-like compounds consisting of amino acids. In order to test this assumption soluble HLA-DR1 molecules were incubated with the high-affinity peptide ligand HA306-318 in the presence of amino acids or very short peptides. While free amino acids did not exhibit any effect (data not shown) the simple dipeptide Tyr-Arg (YR) accelerated the MHC-loading with the HA306-318 peptide in a dose-dependent way (Fig. 1B). No effect was observed with the Ala-Arg (AR) dipeptide lacking the aromatic anchor side chain, indicating that the replacement of tyrosine by alanine completely abrogated the MLE-effect.

**Figure 2:**
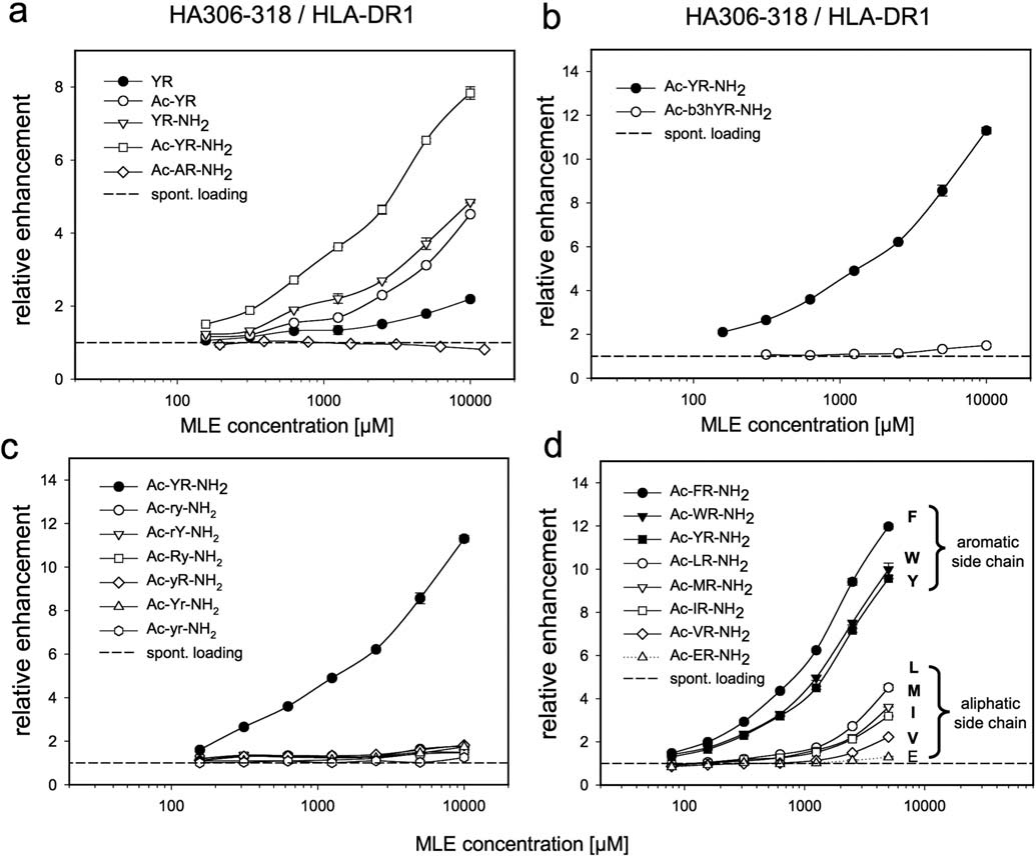
Structure/activity relationships of catalytic dipeptides. a) Role of H-bonds for the catalytic activity of dipeptides. Various H-bond bridges proximal to the P1 pocket stabilize the ligand complex (compare Fig. 1a). N-terminal acetylation and C-terminal amidation was introduced to the YR dipeptide to facilitate the utilization of this H-bond network by minimal peptide-MLE catalysts. The influence was demonstrated in loading reactions with HA306-318 and sol. HLA-DR1 in the presence of titrated amounts of YR (filled circle), Ac-YR (open circle), YR-NH_2_ (open triangle), Ac-YR-NH_2_ (open square) and as control Ac-AR-NH_2_ (open diamond). b) Impact of elongated side chain spacing. A dipeptide derivative was used in which the side chain spacing was elongated a single CH_2_-group by using the L-β-homotyrosine (Ac-b3hYR-NH_2_; open circle) instead of tyrosine (Ac-YR-NH_2_; filled circle). c) Influence of D-amino acids. Complex formation was carried out in the presence of titrated amounts of Ac-YR-NH_2_ (filled circle), Ac-ry-NH2 (open circle), Ac-rY-NH_2_ (open triangle), Ac-Ry-NH_2_ (open square), Ac-yR-NH_2_ (open diamond), Ac-Yr-NH_2_ (open triangle up), Ac-yr-NH_2_ (open hexagon). D-amino acids are indicated by small letters. d) Structural requirements of the catalytic anchor side chain. The P1 pocket of HLA-DR1 interacts preferably with bulky hydrophobic anchor side chains. To compare the catalytic activity with the known structural preferences of P1 the complex formation of HA306-318/HLA-DR1 is shown for Ac-FR-NH_2_ (filled circle), Ac-WR-NH_2_ (filled triangle down), Ac-YR-NH_2_ (filled square), Ac-LR-NH_2_ (open circle), Ac-MR-NH_2_ (open triangle down), Ac-IR-NH_2_ (open square), Ac-VR-NH_2_ (open diamond), Ac-ER-NH_2_ (open triangle up).

As illustrated in Fig. 1A the stability of the MHC/ligand complex is maintained by an H-bond network formed with the backbone of the peptide. A considerable number of these bonds is formed in the immediate vicinity of the P1-pocket. To further stabilize the dipeptide by maximizing the number of H-bonds, acetyl- and amide-groups were introduced to the N- and C-termini of the dipeptides. Computational docking of acetylated dipeptide amide Ac-YR-NH_2_ to the P1-pocket of HLA-DR1 indicates that as much as five of the conserved H-bonds can be formed with this minimal peptide (coordinates of the docking is enclosed in the supplemental PDB-file PDB_Coordinates S1). Compared to the free dipeptide YR a more than 10 fold increase in the catalytic activity was observed with Ac-YR-NH_2_ (Fig. 2A). A partial MLE-effect was observed also with Ac-YR and YR-NH_2_, whereas Ac-AR-NH_2_ was still completely inactive. To confirm the importance of a natural peptide structure, a dipeptide analogue to Ac-YR-NH_2_ was employed in which tyrosine was replaced by β-homotyrosine (b3hY). The introduction of this amino acid increased the distance between the side chains by an additional CH_2_-group and resulted in a total loss of activity (Fig. 2B). Likewise any replacement of the standard L-amino acids by the respective D-enantiomer abrogated the catalytic activity. Also the dipeptide Ac-ry-NH_2_ composed of D-amino acids in inverse sequence did not show any effect, indicating a strict stereo-specificity of the catalyst (Fig. 2C).

Steric requirements, H-bond usage and in particular the failure of dipeptides lacking the aromatic side chain further supported the assumption that the effect was mediated by the dimorphic P1 pocket. The pocket of HLA-DR1 (DRB1*0101) contains the residue β86G, which results in a preference for aromatic and, to a lower extent, for aliphatic anchor residues [14]. To determine whether these preferences are reflected in the catalytic activity of peptide-MLE, a collection of acetylated dipeptide amides was tested in which the tyrosine residue of Ac-YR-NH_2_ was replaced by one of the two other aromatic amino acids phenylalanine (F) and tryptophan (W) and by the aliphatic amino acids leucine (L), methionine (M), isoleucine (I), and valine (V). In line with expectation, strongest increase was observed with the dipeptides containing F, Y or W, while the dipeptides with aliphatic side chains showed weaker activity (Fig. 2D). No enhancement was detected with Ac-ER-NH_2_ where tyrosine was substituted by glutamate (E) which belongs to the residues not fitting in the P1 pocket of HLA-DR1 [15].

The mean catalytic MLE-activity is summarized in Table 1. For soluble HLA-DR1 (DRB1*0101) the catalytic rate enhancement coefficient was determined to be 6.5 mM^−1^, 3.7 mM^−1^ and 3.5 mM^−1^, for minimal peptide-MLE containing F, Y and W respectively. Compared to Ac-FR-NH_2_, the dipeptides with aliphatic MLE side chains exhibited only between 12% (L) and 4% (V) of the activity. Similar catalytic activity was also detected for some unmodified tripeptides as well as for two peptides derived from invariant chain (LRMK, LRMKLPK) [16]. As ‘Ii-key’ they had been described to facilitate MHC-loading by targeting an allosteric invariant chain binding site located outside the P1 pocket. Their activity, however, was not significantly higher than that of the unmodified tripeptides and the combined use with the more active peptide-MLE Ac-FR-NH_2_ did not show any cooperativity (data not shown). Notably, no effect was observed for the N-terminal fragment of the invariant chain octapeptide (LRKPPKPV). Although the fragment was reported to facilitate antigen-loading and catalyze the self-release of the invariant chain peptide IC106-120 (CLIP) [17] at least in this experimental system no catalytic effect was observed.

**Table 1:** Catalytic activity of short peptides on the loading of soluble HLA-DR1 with HA306-318

	Compound*	Catalytic Rate Enhancement** [×103 M-1 ]	rel. cat. Activity*** [%]
a) Minimal peptide-MLE			
1	Ac-FR-NH_2_	6.5 +/− 1.2	100
2	Ac-YR-NH_2_	3.7 +/− 1.0	57
3	Ac-WR-NH_2_	3.5 +/− 0.9	54
4	Ac-LR-NH_2_	0.76 +/− 0.08	12
5	Ac-MR-NH_2_	0.52 +/− 0.00	8
6	Ac-IR-NH_2_	0.43 +/− 0.01	7
7	Ac-VR-NH_2_	0.25 +/− 0.02	4
8	Ac-ER-NH_2_	0.02 +/− 0.02	0
9	Ac-AR-NH_2_	0.00 +/− 0.00	0
b) Catalytic tripeptides			
10	YFR	0.68 +/− 0.29	11
11	YKT	0.59 +/− 0.12	9
12	KYV	0.51 +/− 0.15	8
13	GYV	0.49 +/− 0.16	8
c) ‘Invariant Chain’-derived peptides			
14	LRMKLPK	0.98 +/− 0.21	15
15	LRMK	0.53 +/− 0.15	8
16	LRKPPKPV	0.00 +/− 0.00	0

* ‘Minimal peptide-MLE’ and ‘catalytic tripeptides’ are introduced in this study, catalytic activity for ‘invariant chain derived peptides’ has been reported for LRMK and LRMKLPK [16] and for LRKPPKPV [17].

** The ‘Catalytic Rate Enhancement’ coefficient (*k*) represents the relative increase of the spontaneous loading rate (r_spont_) in the presence of the catalytic peptide (P_cat_). The total rate (r_tot_) can be calculated by (r_tot_ = r_spont_+*k* [P_cat_] r_spont_).

*** “rel. cat. Activity” indicates the relative catalytic activity of peptide derivatives and is expressed as percentage in reference to the catalytic rate enhancement of Ac-FR-NH_2_.

**Figure 3:**
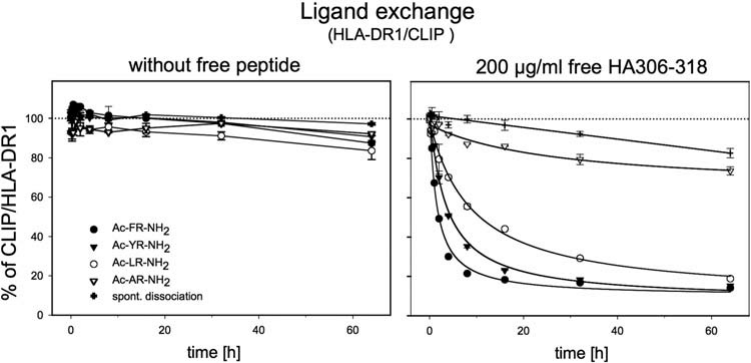
Catalytic dipeptides trigger reversible ligand exchange. To demonstrate that peptide-MLE can catalyze the reversible ligand exchange, complex dissociation of CLIP/HLA-DR1 induced by the peptide-MLE was determined in the absence (left panel) or presence of 200 µg/ml free HA306-318 (right panel). In this experiment 10 mM Ac-FR-NH_2_, (filled circle), Ac-YR-NH_2_ (filled triangle), Ac-LR-NH_2_ (open circle), Ac-AR-NH_2_ (open triangle) or no catalysts (cross) was used. The percentage of CLIP/HLA-DR1 complex remaining after indicated time points was determined by ELISA.

So far, the impact of peptide-MLE was determined only on the loading of empty MHC molecules. To evaluate their influence on complex dissociation soluble class II MHC molecules were preloaded with the medium affine CLIP peptide (Fig. 3). Although the peptide binds to HLA-DR1 with lower affinity than HA306-318 there was virtually no spontaneous decay detectable (left panel). The same applies also when the experiment was carried out in the presence of dipeptide-MLE. The situation looked different when HA306-318 peptide was added (right panel). In less than 2 h, 50% of the HLA-DR1/CLIP complex disappeared when Ac-FR-NH_2_ was present during dissociation. Notably, in the absence of catalyst more than 80% of the complex still remained intact even after 64 h of incubation indicating that the dipeptide-MLE is able to increase also the off-rate of peptide-ligands. In the presence of Ac-YR-NH_2_ and Ac-LR-NH_2_ the half-life was shortened to <4 h and <10 h while Ac-AR-NH_2_ had only a very limited effect. The reversible acceleration of both complex formation and complex dissociation could therefore be correlated with the anchor preference of the P1 pocket.

**Figure 4:**
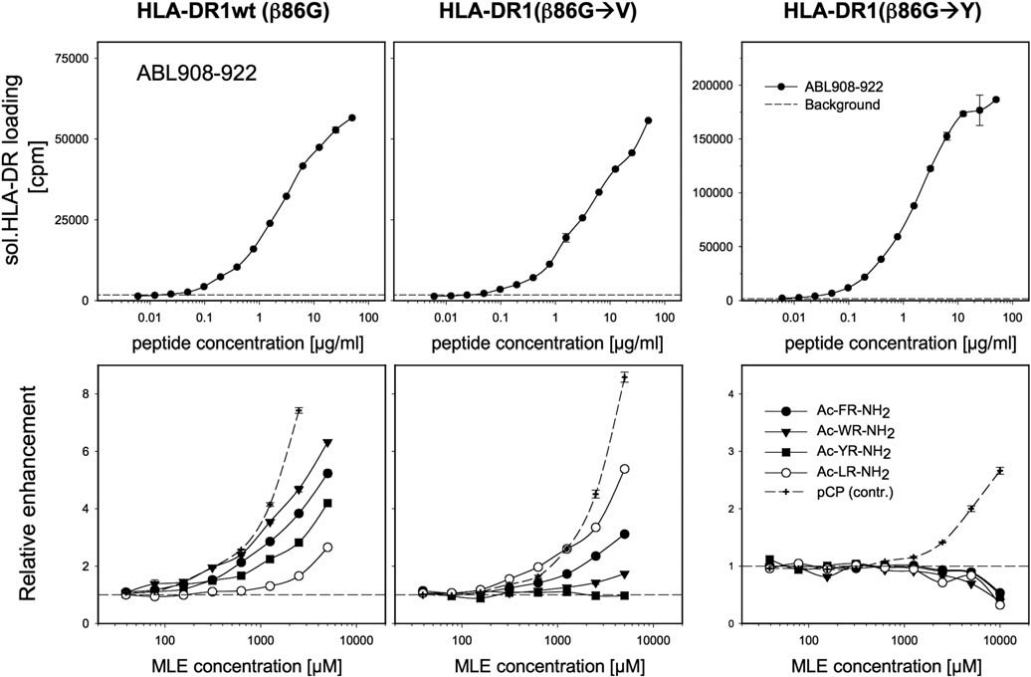
Allele selectivity of catalytic dipeptides. Recombinant soluble HLA-DR1 molecules were mutated inside the P1 pocket and used in loading experiments with ABL908-922, a peptide able to bind to wt as well as to the mutated forms of HLA-DR1 (S. Höpner, unpublished). Upper panels: the spontaneous loading of ABL908-922 is shown for wt HLA-DR1 (β86G), for HLA-DR1 (β86G→V) and HLA-DR1 (β86G→Y). The formation of ABL908-922/HLA-DR complex is expressed in counts per minute (cpm); dashed line indicates background signal. Lower panels: The allele-selective effect of catalytic dipeptides is shown. The influence on HLA-DR loading is shown for Ac-FR-NH_2_ (filled circle), Ac-WR-NH_2_ (filled triangle down), Ac-YR-NH_2_ (filled square) and Ac-LR-NH_2_ (open circle) and for p-chlorophenol (pCP; cross with dashed line), a simple aromatic MLE compound acting independent of P1 [13]. 1.5 µg/ml ABL908-922 were used for wt HLA-DR1 and HLA-DR1 (β86G→V) and 0.2 µg/ml for HLA-DR1 (β86G→Y). Complex formation is expressed as relative enhancement in reference to the spontaneous complex formation in the absence of any catalyst. The loading was determined by ELISA.

In order to formally demonstrate that the catalytic side chains of peptide-MLE act through the P1 pocket, mutants of HLA-DR1 were generated in which the glycine residue β86 at the floor of P1 was replaced either by valine (β86V) or by tyrosine (ß86Y) (Fig. 4). In HLA-DR, β86V represents the natural dimorphic alternate to β86G. Occupation of β86 by valine produces a shallow hydrophobic pocket that is able to accommodate aliphatic side chains but, in contrast to β86G-pockets, is too small for bulky aromatic residues [18]. The non-natural substitution β86Y has been shown to produce MHC molecules that are highly receptive but contain a P1 pocket blocked by the tyrosine residue [19]. Here, only those peptides can bind where the binding does not depend on the P1 pocket. Therefore, loading experiments were therefore carried out with ABL908-922, a pentadecapeptide derived from the ABL kinase that can form a complex with all three variants (Höpner et al. manuscript in preparation) (Fig. 4, upper panels). In the absence of any peptide-MLE, ABL908-922 binds equally strong to wt HLA-DR1 (β86G) and HLA-DR1 (β86G→V). Due to the inherently increased receptiveness of the mutant [19], it exhibits the highest spontaneous on-rate with HLA-DR1 (β86G→Y). Also here the addition of peptide-MLE resulted in increased loading reactions (Fig. 4, lower panel). With wt HLA-DR1 (β86G) the enhancement of ABL908-922 loading corresponded to the result obtained with HA306-318, in which the aromatic dipeptides showed stronger enhancements than the aliphatic Ac-LR-NH_2_ peptide. The pattern was reversed for HLA-DR1 (β86G→V). In line with the anchor preferences of the shallow P1 pocket, best enhancement was obtained with Ac-LR-NH_2_ while weaker amplification was detected with aromatic dipeptides. No enhancement was observed with HLA-DR1 (β86G→Y). Even a slight reduction was detected at the highest dipeptide concentration used, while 4-chlorophenol (pCP), a simple disubstituted benzene known to act independent of P1 [12], [13], still exhibited an MLE-effect.

**Figure 5:**
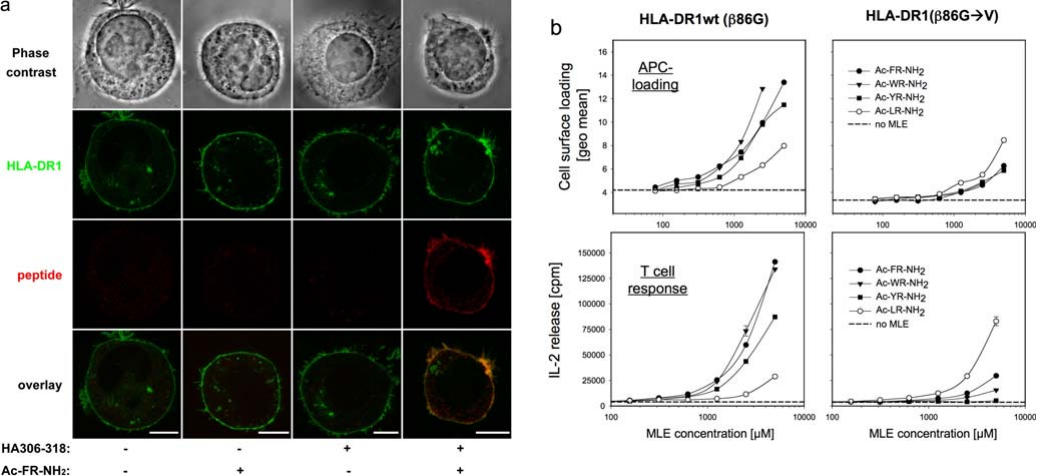
Enhanced loading of cell surface MHC by peptide-MLE. a) Confocal laser scanning analysis of cell surface loading. 721.221-DRb1GFP cells expressing a GFP-tagged HLA-DR1 molecule were incubated with biotinylated HA306-318 peptide in the absence or presence of Ac-FR-NH_2_. After staining with streptavidin-Cy5 images were taken by confocal laser scanning microscopy. Scale bar represents 10 µm. b) Impact on APC loading and T cell response. Fibroblast transfectants expressing either wt HLA-DR1 (left panels) or mutated HLA-DR1 (β86G→V; right panels) were incubated with ABL908-922 in the presence of titrated amounts of Ac-FR-NH_2_ (filled circle), Ac-WR-NH_2_ (filled triangle), Ac-YR-NH_2_ (filled square), Ac-LR-NH_2_ (open circle) or in the absence of any peptide-MLE. Upper panels: Analysis of cell surface loading by FACS. Fibroblast cells were incubated with 12 µg/ml biotinylated ABL908-922. After 4h peptide loading was determined by FACS and is expressed as geometrical mean (geo. mean). Lower panels: Enhancement of the ABL908-922-specific T cell response. Fibroblast cells expressing wt HLA-DR1 or HLA-DR1 (β86G→V) were incubated for 4 h with 150 ng/ml or 300 ng/ml ABL908-922, washed and used to challenge SaABL1/G2, an ABL908-922 specific T cell hybridoma that recognizes the peptide on both HLA-DR1 molecules. The response is expressed as IL-2 release; dashed lines represent the T cell response triggered in the absence of any MLE compounds.

Exposure of cells to synthetic organic MLE facilitated the antigen loading directly on cell surface MHC molecules [12], [13]. To determine whether this applies also for peptide-MLE, 721.221 cells expressing HLA-DR1-GFP fusion proteins (721.221-DRb1GFP) were incubated in the absence or presence of Ac-FR-NH_2_ with biotinylated HA306-318 peptide. After staining with fluorescence-labelled streptavidin, imaging of the cells by confocal laser scanning microscopy revealed a striking increase in the amount of peptide bound to the cell surface in the presence of Ac-FR-NH_2_ (Fig. 5A). While almost no HA306-318 peptide was detectable when the loading was carried out in the absence of the dipeptide-MLE, the addition of the catalyst resulted in a bright surface staining that colocalized with the HLA-DR1-GFP fusion protein.

In a more detailed analysis the peptide loading of cells was analyzed by FACS (Fig. 5B). For these experiments fibroblast cells were used that do not express endogenous class II MHC molecules but were transfected with full length versions of wt HLA-DR1 (β86G) and mutated HLA-DR1 (β86G→V). As in the previous experiment the cells were incubated with biotinylated MHC-binding peptide ABL908-922 in the absence or presence of titrated amounts of catalytic dipeptides (Fig. 5B, upper panels). Quantification of peptide-loading by FACS revealed a similar pattern as observed before with soluble MHC molecules (compare Fig. 4). On fibroblasts expressing wt HLA-DR1 the strongest enhancement was observed with aromatic dipeptides, while the aliphatic Ac-LR-NH_2_ peptide showed the weakest effect. Similar effects were also observed with bone-marrow derived dendritic cells obtained from HLA-DR1 transgenic mice (data not shown). On cells expressing the mutated β86G→V molecule, the aliphatic Ac-LR-NH_2_ was more effective than the peptide-MLE with aromatic side chains. Thus, also the enhancement cell surface loading correlated with the allele-specific anchor preferences of P1.

Importantly, the increased loading efficiency translated directly into improved CD4+ T cell responses (Fig. 5B, lower panels). CD4+ T cell hybridoma specific for the ABL epitope (SaABL/G2) showed the strongest response when they were stimulated with fibroblast cells that had been loaded before in the presence of the dipeptides. The pattern of enhancement reflected the catalytic effect on antigen loading as it directly correlated with the allele-specific P1-anchor preference. Aromatic dipeptides were most efficient on wt HLA-DR1 (β86G) while the aliphatic Ac-LR-NH2 showed best stimulation with cells expressing the mutated HLA-DR1 (β86G→V) with the shallow P1 pocket.

**Figure 6:**
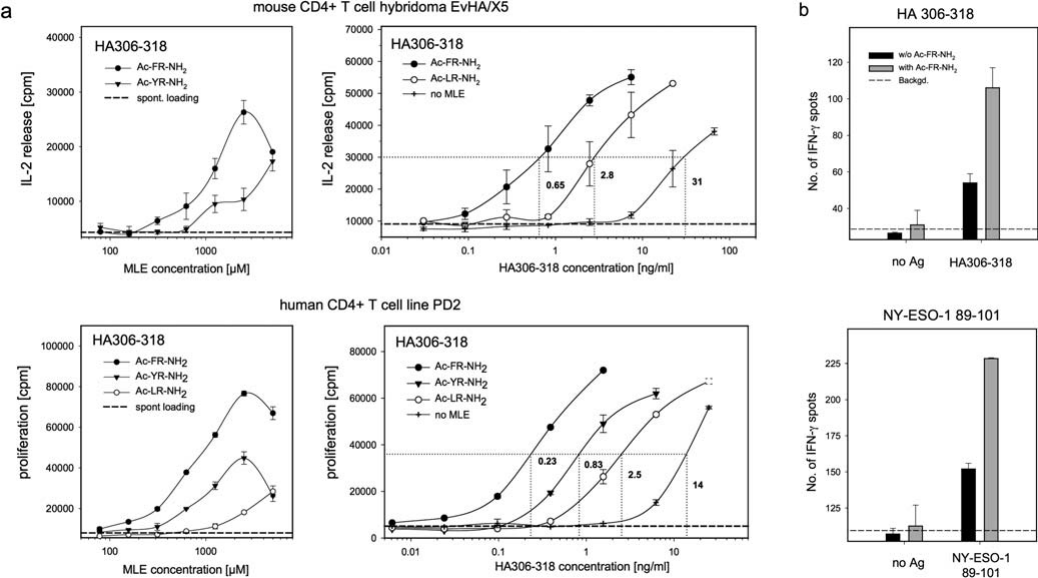
Amplification of the antigen-specific T cell response. a) Enhancement of the *in vitro* T cell response. The influence of catalytic peptides on the antigen-specific CD4+ T cell response was tested with a mouse T cell hybridoma EvHA/X5 (upper panels) and a human T cell line PD2 (lower panels). Both recognize the HA306-318 antigen in the context of HLA-DR1. The left panels show the influence of titrated amounts of Ac-FR-NH_2_ (filled circle), Ac-YR-NH_2_ (filled triangle) or Ac-LR-NH_2_ (open circle) by EvHA/X5 and PD2 in the presence of 15 or 2 ng/ml HA306-318, respectively. The response in the absence any catalyst is indicated as a dashed line. The right panels are showing the dose response curves of HA306-318 in the presence of 5 mM Ac-FR-NH_2_ (filled circle) or Ac-LR-NH_2_ (open circle), 3 mM Ac-YR-NH_2_ (filled triangle) or in the absence of any peptide-MLE (cross). Dashed line indicates the background. b) Enhancement of the *ex vivo* T cell response. Lymph node cells were isolated from HLA-DR1tg mice primed with HA306-318 peptide or NY-ESO-1 89-101. The *ex vivo* response was determined by an IFN-γ ELISPOT assay by challenging the cells with 5 ng/ml HA306-318 (upper panel) or 50 ng/ml NY-ESO-1 89-101 (lower panel), respectively. The bars represent the number of spots detected in the absence (black bar) or presence of 2.5 mM Ac-FR-NH_2_ (grey bar). Each spot originates from a single IFN-γ secreting cell; dashed line indicates the background signal.

The influence of catalytic dipeptides on the CD4+ T cell response was further studied in *in vitro* T cell assays in which T cells and APC were exposed to free peptide antigens and peptide-MLE. In these experiments HA306-318 was used as antigen to challenge two different HLA-DR-restricted CD4+ T cells, the mouse T cell hybridoma EvHA/X5 (Fig. 6A, upper panels) and the human T cell line PD2 (Fig. 6A, lower panels). For both cell lines the presence of peptide-MLE resulted in a drastic increase in the sensitivity of the T cell response. Titration of the peptide-MLE at suboptimal antigen dosage revealed maximal enhancement of the T cell response at concentrations around 2–3 mM (left panels). At these concentrations the dose response curves for the HA306-318 antigen were shifted up to 50-fold towards lower concentrations (right panels). While a half-maximal response in the absence of catalyst was detected at a concentration of 31 ng/ml for EvHA/X5 and of 14 ng/ml for PD2, Ac-FR-NH_2_ lowered the threshold to 0.65 ng/ml and 0.23 ng/ml, respectively. In line with the previous data weaker effects were determined with Ac-LR-NH_2_.

Lastly, to confirm that peptide-MLE mediated enhancement can be observed also with primary cells, lymph node cells from HLA-DR1tg mice were challenged *ex vivo* in the absence or presence of Ac-FR-NH_2_. The mice were primed either with HA306-318 or with NY-ESO-1 89-101, a CD4+ T cell epitope derived from the NY-ESO-1 protein associated with various solid tumours [20]. After 12 days the antigen-specific *ex vivo* response was determined by an IFN-γ ELISPOT assay (Fig. 6B). In line with the previous results, the catalytic dipeptide was found to significantly increase the sensitivity of the assay. At concentrations of 5 ng/ml HA306-318 (upper panel) and of 50 ng/ml NY-ESO-1 89-101 (lower panel) the number of spots representing single IFN-γ secreting cells was significantly higher when the Ac-FR-NH_2_ was added. Thus, short peptides exhibiting MLE-like activity can amplify immune responses also in primary cultures containing ‘natural’ CD4+ T cells and professional APC.

## Discussion

Our experimental data show that short peptide fragments can influence the ligand composition of class II MHC molecules in a catalytic way. By placing an amino acid side chain into a defined pocket of the MHC molecule they trigger ligand-exchange and antigen-loading. Mutational analysis indicated already that the occupation of pocket P1 is crucial for the catalytic effect of organic MLE compounds [12]. As demonstrated here for the human molecule HLA-DR1, a similar role could also be established for the ligand exchange driven by short peptides. P1 is present in all MHC class II molecules. It is located within the peptide binding site and accommodates the side chain of a key-anchor residue of the peptide ligand. While the location of the pocket is conserved, it contains polymorphic residues that determine allele-specific preferences for anchor residues. Since the same structural requirements also dictate the interaction with ‘catalytic’ side chains of short peptides, they exhibit their effect in an allele-selective way.

As shown before for simple organic compounds the MLE mechanism is based on the stabilization of the peptide-receptive conformation [12]. Earlier studies showed already that the substitution of residue β86G by tyrosine resulted in a ‘filled’ P1 pocket and produced an MHC molecule with elevated receptiveness [21]. P1 is located proximal to the binding site of HLA-DM and binding studies suggested that HLA-DM interacts specifically with the flexible empty hydrophobic P1-pocket [21]. While the active conversion of a non-receptive molecule by HLA-DM has recently been questioned [22], it is undisputed that the chaperone interacts with a region proximal to P1 to stabilize the peptide receptive conformation [10], [11], [23], [24].

**Figure 7:**
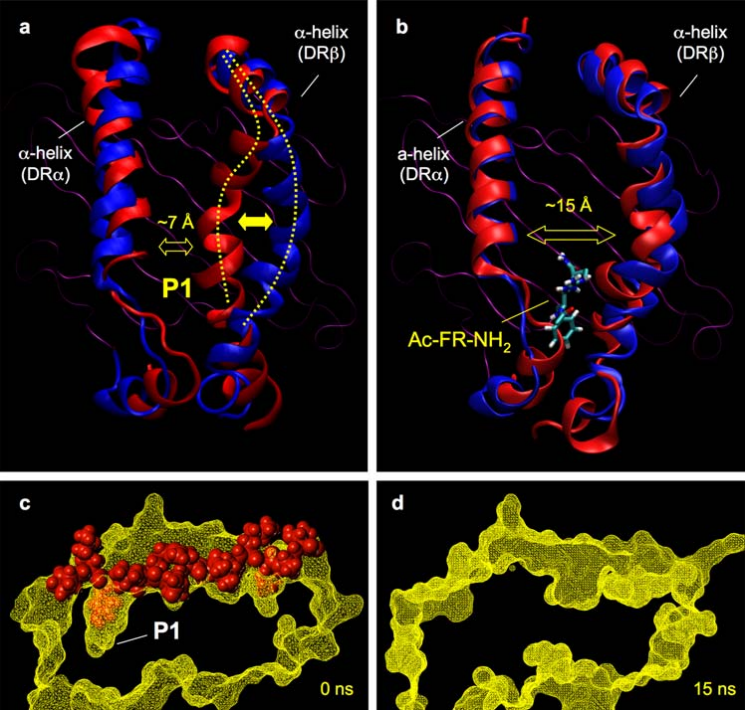
Molecular dynamic (MD) calculation of ‘empty’ and peptide-MLE stabilized HLA-DR1. The coordinates of the MHC component of the crystallized HLA-DR1/HA306-318 complex (1DHL) were used to carry out a 15 ns MD calculation with an ‘empty’ MHC molecule. a) Dynamic of the empty MHC molecule. The floor composed of the β-plated sheats is depicted in magenta, the α-helices of the starting structure are shown in red, α-helices of the structure obtained after 15 ns are shown in blue. The approximate position of P1 is indicated. While the dynamic was carried out with all extracellular domains, only the binding site is shown (α_1_-, β_1_-domain). b) Dynamic of the peptide-MLE stabilized MHC molecule. The same MD calculation was carried out as in Fig. 7a except that coordinates of an HLA-DR1 molecule were used, in which prior to the MD calculation the peptide-MLE Ac-FR-NH_2_ was docked into the P1 pocket. c) P1 pocket in the peptide loaded MHC complex. The image shows a cross-section of the HLA-DR1/HA306-318 complex. The surface of the MHC molecule is shown in yellow, the peptide ligand in red; position of the P1 pocket is indicated. d) Loss of P1 in the empty MHC molecule. The same cross-section shown in Fig. 7c for the peptide-loaded MHC is shown here for the empty molecule obtained after 15 ns of MD calculation. In this structure the P1 pocket can no longer be located.

In a recent publication we introduced a model in which the transition to the non-receptive state is directly correlated with structural changes inside pocket P1. Experimental evidence was taken from the observation that P1-targeting MLE compounds prevent this transition [12]. The strict correlation of the catalytic activity with the structural requirements of P1 introduced by this study provides additional support to the hypothesis that the stabilization of P1 prevents the transition into the non-receptive state. A molecular dynamic (MD) simulation confirmed that the pocket P1 is indeed quickly lost when the peptide ligand is stripped off from the MHC molecule (Rupp et al. manuscript in preparation) (Fig. 7). Calculations based on the coordinates of the crystal structure of the HLA-DR1/HA306-318 complex revealed that the most significant transitions were detected near the P1 pocket. While these shifts resulted in a narrowing of the two α-helices by more than 7Å (Fig. 7A), they also led to a complete loss of the P1 pocket. In less than 15 ns the P1 cavity was filled with side chains or removed by distortions (Fig. 7C,D). Notably, this collapse was prevented when prior to the MD simulation the Ac-FR-NH_2_ was docked into the P1 pocket (Fig. 7B).

Based on this model even the partial occupation of the binding site by a very short peptide is sufficient to stabilize the receptive state as long as it positions an anchor side chain inside the P1-pocket (supplemental PDB file PDB_Coordinates S1). P1 therefore seems to function as a sensor for the peptide load where occupation leads to a stabilization of the ‘open’ conformation required to accommodate the peptide ligand. Studies by other groups have already shown that the loading status of P1 plays a crucial role as indicator in the intracellular antigen-processing pathway. The interaction with HLA-DM seems to depend on the loading state [21] and its catalytic activity was reported to be mediated by βH81, a conserved residue located on top of the P1 pocket [24].

While inside the cell the occupational state of P1 seems to control the interaction with key-components of the processing pathway, on the cell surface it may regulate the transition into the non-receptive conformation. Here, it functions as trigger for a safeguard mechanism that closes the binding site as soon as the ligand is lost. In this study we showed that small peptide fragments can by-pass this mechanism in a catalytic way. Particularly striking is the effect on the ligand exchange. Peptide-MLE were able to increase not only the loading of empty HLA-DR molecules but also the dissociation of HLA-DR molecules preloaded with lower-affinity ligands in a reversible reaction. Both effects should account for the increased antigen-loading of cell surface MHC molecules, which translated directly into improved CD4+ T cell responses. As molecular tool MLE compounds may therefore find applications in experimental and therapeutic settings in which improved antigen loading is desired. A particular suitable field may be peptide-based tumour immune interventions, where the exposure of antigen to a hostile proteolytic environment is extended by the limited access to receptive MHC molecules on the surface of professional APC.

While the importance of CD4+ T cells for productive tumour immune responses has just begun to be fully discovered [25] their role in the induction of autoimmune responses has long been acknowledged [26]. It is evident for instance in the strong genetic link to class II MHC molecules and in the fact that experimental autoimmune diseases can often be induced by the adoptive transfer of auto-aggressive CD4+ T cells. In this respect ‘accidental’ loading of these cells with self-antigens by peptide-MLE may therefore trigger unwanted auto-aggressive responses. *In vitro* we have shown already that the presence of simple organic MLE compounds can enhance encephalitogenic T cell responses [12], [13]. The same may also apply for colitis or celiac disease. Intestinal dendritic cells are known to penetrate gut epithelia cells [27] and expose their dendrites inside the gut lumen to extremely high polypeptide concentrations originating from the diet or commensal debris. Capture of soluble antigens by immature DC from lymph or blood, on the other hand, also seems to be an important mechanism for tolerance induction [28] and direct cell surface loading has been discussed as an alternative processing pathway of immature dendritic cells [6], [7]. Natural protein fragments present in blood or lymph acting as peptide-MLE may therefore participate in this process by mediating the direct transfer of antigens onto cell surface MHC molecules.

## Materials and Methods

### Compounds and reagents

The following peptides were used: IC106-120 (KMRMATPLLMQALPM; ‘CLIP’ peptide) [29], HA306-318 (PKYVKQNTLKLAT) [30], NY-ESO-1 89-101 (EFYLAMPFATPME) [20] and human ABL 908-922 (KGKLSRLKPAPPPPP) (Hopner et al., unpublished). N-terminal biotinylation was introduced using two 6-amino hexanoic acid spacer units. Stock solutions of short peptides (100 mM) were prepared with DMSO/PBS after ultrasound sonication at the following DMSO concentrations: YR, AR (0%); LRMK, YFR, GYV, Ac-AR-NH_2_, Ac-ry-NH_2_, Ac-rY-NH_2_, Ac-Ry-NH_2_, Ac-yR-NH_2_, Ac-Yr-NH_2_ and Ac-b3hYR-NH_2_ (10%); Ac-FR-NH_2_, Ac-YR-NH_2_, Ac-LR-NH_2_, Ac-ER-NH_2_ (15%); LPKPPKPV (20%); Ac-WR-NH_2_ (25%); Ac-VR-NH_2_, Ac-MR-NH_2_, Ac-IR-NH_2_, Ac-RY-NH_2_ (100%). All peptides were produced by EMC microcollections GmbH (Tübingen, Germany) and analyzed by RP-HPLC (214 nm) and ESI-MS.

### Antibodies and soluble HLA-DR1

Phycoerythrin- (PE) conjugated streptavidin was purchased from Caltag, α-HLA-DR-PE (L243) was obtained from BD Biosciences. Unlabelled α-HLA-DR (L243) and α-IFN-γ (AN18.1724 and R4-6A2) were purified from hybridoma supernatant by Prot.A and Prot.G columns (GE Healthcare). R4-6A2 was labeled with NHS-Biotin according to the manufactures recommendation (Pierce). Soluble wt HLA-DR1 (DRA*0101, DRB1*0101) [31] was produced in S2 insect cells as described [13]. Mutant forms of HLA-DR1 were expressed in a baculovirus expression system. Briefly, DNA coding for the extracellular domains of DRA*0101 and DR1B*0101 was separately cloned into the transfer vector pFastbac 1 (Invitrogen). Leucine zipper domains were added to the C-termini of the α- and β-chain as described [32]. Site-directed mutagenesis of HLA-DR1 β-chain was carried out using the QuickChange site-directed mutagenesis kit (Stratagene). Recombinant viruses were generated in *S. frugiperda* cells (Sf21). For expression of proteins, cells were co-infected with viruses for the α- and β-chain.

### Cells

The following class II expressing cell lines were used: L929 fibroblasts (ATCC) transfected with wt (DRB1*0101) or mutated HLA-DR1 (HLA-DRB1*0101 β86V) [12], EBV-transformed B cell 721.221 (ATCC) and HTR [33]. 721.221-DRb1GFP cells were produced by stably transfecting 721.221 cells with a HLA-DR ß-chain (DRB1*0101) C-terminally fused to EGFP (Falk et al. unpublished). The following T cells were used: DRB1*0101-restricted, HA306-318-specific mouse hybridoma line EvHA/X5 [12] and human CD4+ T cell line PD2 [33]; the ABL 908-922-specific T cell hybridoma SaABL/G2 was generated after fusing a CD4+ T cell line generated in HLA-DR1 tg mice [34] with BW cells (Hopner et al., unpublished).

### Peptide loading of soluble HLA-DR1 molecules

Loading experiments with soluble MHC molecules were carried out as described [13]. Briefly, 100 nM HLA-DR1 was incubated with 50 µg/ml of biotinylated HA306-318 peptide (PBS, pH 7.4, 37°C, 1 h). The amount of peptide/MHC complex formed was determined by ELISA with the α-HLA-DR capture antibody (L243, ATCC) and Eu^3+^-labelled streptavidin (DELFIA,Wallac) using a Victor 3V reader (Perkin Elmer). Ligand exchange experiments were carried out with preloaded HLA-DR1/CLIP complexes (1,5 µM HLA-DR1, 50 µg/ml biotinylated CLIP, 18-20 h, pH 7.4, 5% ethanol/PBS) diluted 1:15 and incubated with or without 200 µg/ml HA peptide in presence and absence of 10 mM dipeptide [12]. All experiments are carried out at 2 or 3 times.

### Peptide loading of cell surface MHC molecules

Loading experiments were carried out as described [12]. Briefly, 1×10^5^ HLA-DR expressing cells/well were incubated with biotinylated MHC-binding peptides in presence and absence of catalytic dipeptides (4 h, 37°C, DMEM, 5% FCS, 96 well V-bottom plates). For FACS analysis cells were stained with streptavidin-PE and analyzed on a FACScalibur instrument (BD Biosciences). Dead cells were excluded by propidium iodide staining. Experiments were carried out twice.

### Confocal laser scanning microscopy

Briefly, 1×10^5^ HLA-DR1 expressing cells (721.221-DRb1GFP) per well were incubated with 20 µg/ml of biotinylated HA 306-318 peptide in presence and absence of 2.5 mM Ac-FR-NH_2_ (4 h, 37°C, DMEM, 5% FCS, 96 well V-bottom plates). Cells were then washed and were incubated on poly-L-lysine (sigma) coated plates for 30 min. at 37°C in RPMI medium (Gibco), followed by a 15 min. 3.7% formaldehyde fixation. After washing cells were stained with streptavidin-Cy5 (Amersham) and mounted with vectashield (vector labs). Fixed cell microscopy was performed with a Zeiss LSM510Meta confocal setup (63× phase contrast plan-apochromat oil objective). Experiments were carried out twice.

### T cell assays

T cell assays were carried out as described [13]. Briefly, 5×10^4^ HLA-DR expressing cells/well were incubated with MHC-binding peptides in presence and absence of catalytic dipeptides (37°C, DMEM, 5% FCS, 96 well round-bottom plates). In assays with antigen-pulsed APC, cells were washed after 4 h before 5×10^4^ T cells were added, in permanent exposure assays the T cells were added directly without prior removal of the peptides. In experiments with T cell hybridoma the culture supernatant was removed after 24 h and the T cell response was determined by measuring IL-2 release in a secondary assay with CTLL cells (ATCC) as described previously [35]. In experiments with T cell lines, APC were radiated with 60.7 Gy and ^3^H-thymidine was added after 48h and the incorporation was determined using a 1450 Microbeta counter (Wallac). Experiments were carried out twice.

### Detection of ex vivo response by ELISPOT assay

HLA-DR1tg mice were primed with 5 µg HA306-318 or 10 µg NY-ESO-1 89-101 in incomplete Freud's adjuvant (Sigma)/50 µg CpG OND 1826 (BioTez GmbH). On day 12, lymph node cells were isolated and incubated in ELISPOT-plates (Multiscreen HTS 96 well Filteration plate; Millipore) coated with α-IFN-γ (clone AN18.1724). Cells were incubated at a density of 1×10^6^ splenocytes/well with indicated amounts of HA306-318 and Ac-FR-NH_2_ peptide (20–40 h, 37°C, 5% CO_2_, RPMI 5% FCS). Detection was carried out according to manufacturer's recommendation using the biotinylated α-IFN-γ detection antibody (clone R4-6A2), avidin–HRP enzyme conjugate (Sigma) and 3,3 diaminobenzidine tablets (Sigma). Spots were counted using ‘Immunospot’ reader (C.T.L Europe GmbH). Experiments were carried out twice.

### Calculation of the ‘catalytic rate enhancement’

The ‘catalytic rate enhancement’ coefficient was determined in loading assays with 100 nM soluble HLA-DR1 and 50 µg/ml HA306-318 with titrated amounts of the catalytic peptide-MLE (1 h, 37°C, pH 7.4). A curve fit was carried out by a hyperbola regression (f(x) = ax/(b+x)) using the Sigmaplot Version 9.0 software (Systat Software Inc.) and the coefficient was determined by forming the average of the starting slope (a/b) of 2–4 independent experiments.

### Docking and ‘Molecular Dynamics’ calculation

The benzene ring of the phenylalanine residue and the backbone of the Ac-FR-NH_2_ dipeptide were superimposed to the residues Y308 and V309 of the HA306-318 peptide in the HA306-318/HLA-DR1 crystal structure 1DLH, followed by a conformation search with the arginine side chain of the dipeptide to find an optimal orientation. Subsequently, the complex structure was minimized using the Tripos software (SYBYL 7.3, Tripos Inc., St. Louis, USA) and the GROMACS force field [36]. For both, the empty MHC structure and the Ac-FR-NH_2_/MHC-complex, a 15 ns molecular dynamic simulation in the GROMACS force field was performed (Rupp et al., manuscript in preparation). The simulations were done under physiological conditions (0.9% NaCl, 310 K) after equilibration over a period of 500 ps using a positional restraint of 1000 kJmol^−1^nm^−2^. Frames were stored every 5 ps, visualisation of trajectories and arrangement of the figures were realised using VMD [37].

## Supporting Information

PDB_Coordinates S1 The text file contains the calculated coordinates of the complex Ac-FR-NH_2_/HLA-DR1 in PDB-file format. For the docking of Ac-FR-NH_2_ the benzene ring of the phenylalanine residue and the backbone of the dipeptide were superimposed to the residue Y308 (located within the P1 pocket) and V309 of the HA306-318 peptide in the HA-306-318/HLA-DR1 crystal structure 1DLH. Subsequently, the complex was minimized using Tripos and the GROMACS force field. Chain A: HLA-DR1 α-chain (DRA*0101); chain B: HLA-DR1 β-chain (DRB1*0101); chain C: Ac-FR-NH_2_
(0.26 MB TXT)
